# The Potential of Research Drawing on Clinical Free Text to Bring Benefits to Patients in the United Kingdom: A Systematic Review of the Literature

**DOI:** 10.3389/fdgth.2021.606599

**Published:** 2021-02-10

**Authors:** Elizabeth Ford, Keegan Curlewis, Emma Squires, Lucy J. Griffiths, Robert Stewart, Kerina H. Jones

**Affiliations:** ^1^Department of Primary Care and Public Health, Brighton and Sussex Medical School, Brighton, United Kingdom; ^2^Swansea Medical School, University of Swansea, Swansea, United Kingdom; ^3^King's College London, London, United Kingdom; ^4^South London and Maudsley NHS Foundation Trust, London, United Kingdom

**Keywords:** clinical free text, natural language processing, text analysis, data governance, privacy, patient benefit

## Abstract

**Background:** The analysis of clinical free text from patient records for research has potential to contribute to the medical evidence base but access to clinical free text is frequently denied by data custodians who perceive that the privacy risks of data-sharing are too high. Engagement activities with patients and regulators, where views on the sharing of clinical free text data for research have been discussed, have identified that stakeholders would like to understand the potential clinical benefits that could be achieved if access to free text for clinical research were improved. We aimed to systematically review all UK research studies which used clinical free text and report direct or potential benefits to patients, synthesizing possible benefits into an easy to communicate taxonomy for public engagement and policy discussions.

**Methods:** We conducted a systematic search for articles which reported primary research using clinical free text, drawn from UK health record databases, which reported a benefit or potential benefit for patients, actionable in a clinical environment or health service, and not solely methods development or data quality improvement. We screened eligible papers and thematically analyzed information about clinical benefits reported in the paper to create a taxonomy of benefits.

**Results:** We identified 43 papers and derived five themes of benefits: health-care quality or services improvement, observational risk factor-outcome research, drug prescribing safety, case-finding for clinical trials, and development of clinical decision support. Five papers compared study quality with and without free text and found an improvement of accuracy when free text was included in analytical models.

**Conclusions:** Findings will help stakeholders weigh the potential benefits of free text research against perceived risks to patient privacy. The taxonomy can be used to aid public and policy discussions, and identified studies could form a public-facing repository which will help the health-care text analysis research community better communicate the impact of their work.

## Introduction

Electronic Health Records (EHRs) are revolutionizing health care at the point of delivery, but also offer huge potential for discovery and research worldwide. The United Kingdom (UK) with its single-payer free-to-access universal health care provision, is particularly well-placed to advance health data science, and the government has invested widely in infrastructure for availability of data, dataset linkage, and analytical capability (1). In the UK, data contained in EHRs is widely used for health research, drug safety analyses, and service planning (2). EHRs have been demonstrated to be a particularly important data source in the UK for conducting epidemiological research within general practice (2–4), mental health (5, 6), hospital episodes (7), and specialist conditions such as cancer (8).

In many cases, data are entered into EHRs in free text natural language in the form of clinic notes, letters and reports. This is particularly true of UK-based mental health records, GP clinic notes and letters, and hospital communications such as pathology or scan reports and discharge letters. These free text natural language data are considered unstructured, in comparison to clinical data stored in preset fields in records or entered in the form of clinical codes, in which a numeric or alphanumeric string represents a unique clinical concept such as a diagnosis or process of care (9). Natural Language Processing (NLP), alongside other methods, can be used to extract information from free text contained within EHRs (10), and therefore, unstructured EHR data can contribute to health research.

However, clinical data and health records are considered highly personal, and thus fall under data protection laws of most jurisdictions, such as the General Data Protection Regulation (2018) in Europe, the UK Data Protection Act (2018) and HIPAA in the USA (11). Data need to be de-identified or anonymized before they can be shared outside the clinical environment for any secondary purposes such as research or service planning or improvement. De-identification of *structured* records happens when fields containing identifiers are stripped out before data-sharing; it is a fairly straightforward process to comply with current data governance regulations. However, de-identifying free text clinic notes, letters and reports is more complex, and is a rapidly evolving field. These data contain names of patients, health-care professionals and family members, they may contain addresses, dates of birth, and other identifying pieces of information, occurring potentially anywhere in the text. Globally, many groups have worked on bespoke algorithms for de-identifying local corpora of free text records using approaches such as rule-based algorithms, pattern matching, conditional random fields, neural networks, recurrent neural networks, and bidirectional transformers (12–19). One drawback of machine learning models is that they require very large marked-up datasets from which to learn, which are not readily available outside of clinical environments, and the lack of available training data hampers progress in this field.

There is a lot of concern that de-identification of free text does not produce a perfect result, and some patient identifiers may slip through. Surveys and reviews have suggested that the best applications work at or above 90% accuracy for all types of personal health information, with systems working best on redacting name, date and age information and worst on profession and ID numbers (20–23). Portability of de-identification algorithms to new datasets is likely to be poor. Many teams now use a “hiding in plain sight” method where redacted identifiers are replaced with plausible substitutes, thus masking the few identifiers which slip through (24). A few studies have assessed the possibility of re-identification of patients from leaks in de-identified free text corpora. Two studies evaluated manual examination of de-identified notes by the original clinicians or by researchers with access to the data, in neither case was any patient correctly re-identified (25, 26). In a third study, using a complex reverse synthesis of de-identification methods, a simulated adversary could have identified 68% of data leaks correctly (27). Authors acknowledged that this mode of attack on de-identified data requires considerable effort and ingenuity from an adversary. At the current time, data custodians have few ways of quantifying or communicating the privacy risks to individuals associated with sharing automatically de-identified data. For this reason, text data which is used for research is often kept behind clinical firewalls or in specially designed data safe-havens.

Because of this lack of clarity of how well privacy can be protected, in the UK, free text notes, letters and reports are generally stripped out of clinical datasets before they are shared for research outside of the clinical environment, for example with university-based research teams. Some teams have created bespoke de-identified free text clinical datasets within clinical firewalls, and bring researchers with NLP skills within the clinical environment to conduct the work (28) but these multi-disciplinary analytics teams are hard to assemble and fund, and are by no means the norm. Within advanced infrastructures, sometimes text data is accessed by sending NLP algorithms into safe-haven data storage and extracting only relevant clinical information in short excerpts or as structured data (29), but usually these algorithms need marked-up training data to develop and evaluate, so do not get round the problem of needing access to full free text entirely.

There has been a concerted, UK-wide effort to clarify policy on sharing or re-using UK clinical free text, through funded research networks such as Healtex, Health Data Research-UK and others. It is clear that a society wide debate is needed on the balance between giving weight to the potential health benefits which could be realized from research analysis of clinical free text data, vs. prioritizing patient privacy. In the research literature and in policy and regulatory discourse around the use of patient data, there has been a notable failure to balance the privacy risks of data-sharing against the ethics of data non-use: not sharing data may be actively harmful, and cost lives, if progress in research is not made (30). We have worked on a portfolio of research to understand the wider context of governance that would be needed for free text data from UK NHS patient records to be shared at scale for research in a way that is acceptable to the public. We have engaged in deliberative research to elicit informed public opinion (31), scoped the data protection landscape and current governance practice for data-sharing, and engaged with other key stakeholders such as researchers, patient representatives, and regulators (32). Throughout our engagement work with UK-based stakeholders, it has been made clear that they want greater understanding of the benefits that improving access to medical free text for research could bring to patients or health-care services. Previous work has shown that adding free text to coded data can improve the accuracy of case detection in research and thus may improve research *quality* (9). But no study has yet brought together the available body of research using free text to produce a resource to describe to relevant stakeholders what the possible benefits to patients might be of conducting research using their clinical free text data, or what harms may be avoided. An understanding of the range of possible benefits in healthcare, achievable in the UK, available from research which uses clinical free text data, would enable stakeholders to weigh benefits against privacy risk when assessing whether they endorse researchers having access to free text data, and contribute to UK-wide policy on sharing of these data.

We aimed to systematically review the literature of studies using clinical free text, conducted using data generated in UK healthcare systems, which reported direct or potential benefits to patients in terms of informing or improving the quality of their care. Given our aim to describe use of free text in the studies and create a taxonomy of potential clinical benefits to aid communication about the reasons for using free text data for research, we used a qualitative approach. This allowed us to synthesize results into an easily communicable set of findings for discussion with key stakeholders in this space and highlight potential case studies. We did not attempt to quantify the level of benefit achieved by the inclusion of free text in data used in the eligible studies.

## Methods

A study protocol was registered on PROSPERO (No. CRD42019141504) (33). The study is reported according to PRISMA reporting guidelines (34).

### Search Strategy

Two systematic database searches were carried out. The first involved a search of articles indexed in PubMed and Web of Science (WoS), conducted in July 2019, using the following search terms:

(1) “electronic health records” or “electronic medical records” or “electronic patient records” or “hospital records” or “personal health records” or “computerized patient records” or “computerized medical records”; combined with (2) “text mining” or “natural language processing” or “free text” or “narrative.”

No limits of date or location were put on this search. The second updated search was performed in March 2020. The same search string defined above was used to search PubMed and WoS. This search was limited to publications after July 2019, in order to update the previous search. Forwards and backwards searching of identified papers led to the identification of the Maudsley Biomedical Research Center (BRC) bibliography of CRIS publications which we examined (35). We further searched the published bibliographies of the Clinical Practice Research Datalink (CPRD) (36), The Health Improvement Network (THIN) (37) and the Secure Anonymized Information Linkage (SAIL) databank (38). All papers identified from searching these bibliographies were included if they met the eligibility criteria.

### Eligibility Criteria

To be eligible for this review, published research had to meet the following criteria: (1) Primary research using free text records, published in English; (2) UK health record databases or hospital data; (3) Information extracted from the text of (human, not veterinary) electronic medical records, medical letters or medical reports; and (4) report a benefit or potential benefit for patients, actionable in a clinical environment (i.e., not solely methods development or data/database quality evaluation). We defined direct or potential benefit as that the study produced a result or finding which could be translated into a change in care delivery or service access or design; or any other action in a clinical environment; which would improve the care for, or outcomes of, patients.

### Screening and Study Selection

Search results were screened first by title, then abstract, then full text against the eligibility criteria. All full text articles were examined and discussed between two authors (KC and one other member of the study team EF, KHJ, ES, or LG) to establish if they met inclusion criteria, where a decision could not be made, a third author arbitrated.

### Critical Appraisal of Included Studies

Because of the heterogeneity of study designs in the included papers we chose the Joanna Briggs Institute (JBI) Critical Appraisal Checklist for Analytical Cross Sectional Studies (39). The articles meeting eligibility criteria above were dual-screened by the authors. Any differences in scoring between the two authors were adjudicated by EF in discussion with KC, until a consensus was reached. Publication bias and selective reporting within studies were not assessed.

### Data Extraction

Characteristics of the included studies were extracted by multiple authors, including author(s), year of publication, country of publication, clinical research question, data source and type, number of patients and number of documents included in analysis, extraction method and purpose of free text in the study, summary of clinical findings, and any statement by authors of actual or potential clinical impact.

### Data Synthesis

Information about clinical benefit was thematically analyzed to create a taxonomy of benefits, according to the following research questions: (1) what are the main types of benefits achieved; (2) are benefits achieved for mental and physical health; and separately (3) is there any evidence that more benefit is achieved by the use or addition of free text compared to structured or coded clinical data?

Firstly, data were identified by authors from the introduction, results or discussion sections which described a direct or potential benefit from the study for patients in terms of improvements to their health or to healthcare provision; these were then copied and pasted into a spreadsheet. The proposed benefits were iteratively examined and grouped (by EF and KC) until authors were content that themes covered all benefits emerging from the data. Papers were then re-read looking for evidence in support of these themes and examining whether they reported on physical or mental health. Themes were then reported in a narrative synthesis; examples from papers are given to support understanding and communication of themes.

Finally, results sections were examined to identify if they reported any statistics showing accuracy or information extraction improvements comparing free text models with models that used only clinical coded data. Where found, these results were reported in a narrative synthesis.

## Results

### Search Results

**Figure 1:**
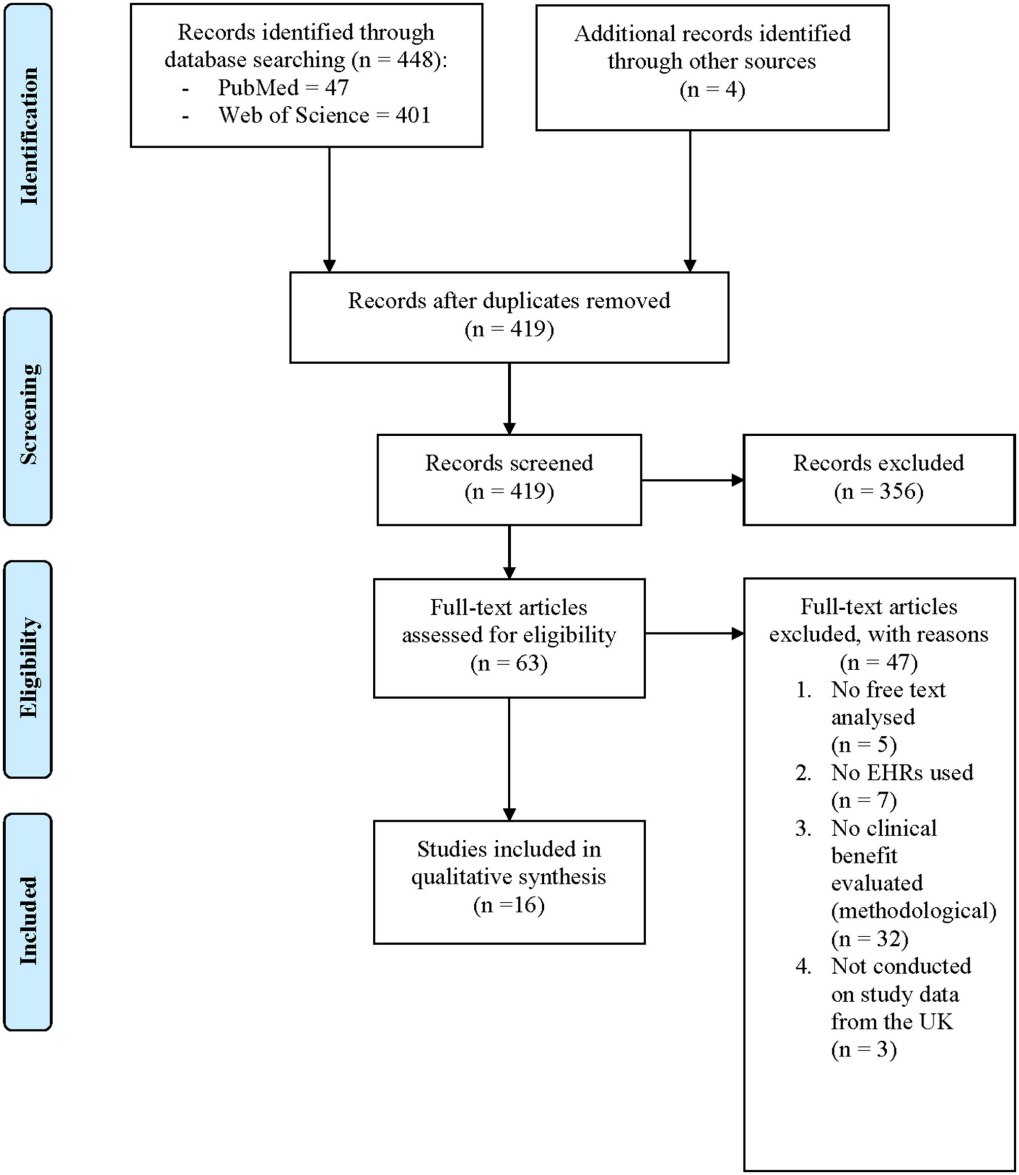
PRISMA flow diagram showing search and screening results.

The two searches identified 448 papers. A further 179 papers were identified from lateral searching of the CRIS online database, no further papers were identified from CPRD, THIN, or SAIL bibliographies. After duplicates were removed, 598 unique papers had their titles and abstracts screened for relevance. Irrelevant papers were excluded, leaving 94 papers that underwent dual full-text review. From these papers, 51 were excluded for reasons displayed in Figure 1. Overall, 43 papers met the full eligibility criteria and were included in this systematic review.

### Study Characteristics

Included papers were published between 2010 and 2020, and all were based on data in the UK as required by inclusion criteria. They analyzed data from South London and the Maudsley (SLaM) Biomedical Research Center (BRC) CRIS database (36 papers), Clinical Practice Research Datalink (CPRD) (four papers), The Health Improvement Network (THIN) (two papers), and hospital patient records (one paper). Details of all included studies are shown in Table A1. All papers met at least four of eight criteria on the JBI Critical Appraisal checklist for analytical cross-sectional studies (final agreed scoring on this tool is supplied in Appendix 1). No papers were rejected on the basis of study quality.

### Analysis and Purpose of Free Text in the Studies

Studies described several methods for extracting information from free text. The simplest form of information extraction was manual review of free text (40–45) or a keyword search for relevant information, which was then modified by an algorithm (46) or manual review (47–49) to check for negation and uncertainty. Shah et al. described a bespoke algorithm for converting general practice text data into categorical data for analysis (50). Papers drawn from the SLAM CRIS database used a bespoke set of data extraction techniques, supplied in the CRIS system using the Generalized Architecture for Text Engineering (GATE) (51) which allows integration of a range of NLP algorithms for specific purposes, such as identification of medications or Mini-Mental State Examination (MMSE) scores for dementia. The Maudsley BRC separately publish a list of clinical concepts which can be extracted from free text by this system and the technical details, specifications and standardized performance metrics for each NLP algorithm (52). Some papers reported software used which overlaid CRIS or GATE, such as TextHunter (53), allowing development of searches for novel clinical concepts. All information extracted from text in these studies was converted to categorical data for statistical analysis.

Free text was used often to supplement ICD diagnosis codes (in the SLAM BRC CRIS case register) and Read codes (in general practice data) to improve identification of patients with the diagnosis of interest, for inclusion in the study (42–44, 54–65). Medication information was often extracted from free text, particularly in studies using the SLaM BRC CRIS case register (57, 60, 63, 64, 66–73). Also extracted from free text were disease symptoms and drug reactions (46, 47, 50, 60, 67, 72, 74–76); test scores, such as for the MMSE (58, 59, 61, 63, 64, 77, 78), and angiogram results (50); treatments such as cognitive behavioral therapy (CBT) (60, 79); substance use behaviors such as cannabis (49, 72, 80), alcohol (43, 44, 49) or smoking status (81); housing status (45); and information on symptom severity and functional status (61, 73).

### Thematic Analysis and Taxonomy of Clinical Benefits

**Figure 2:**
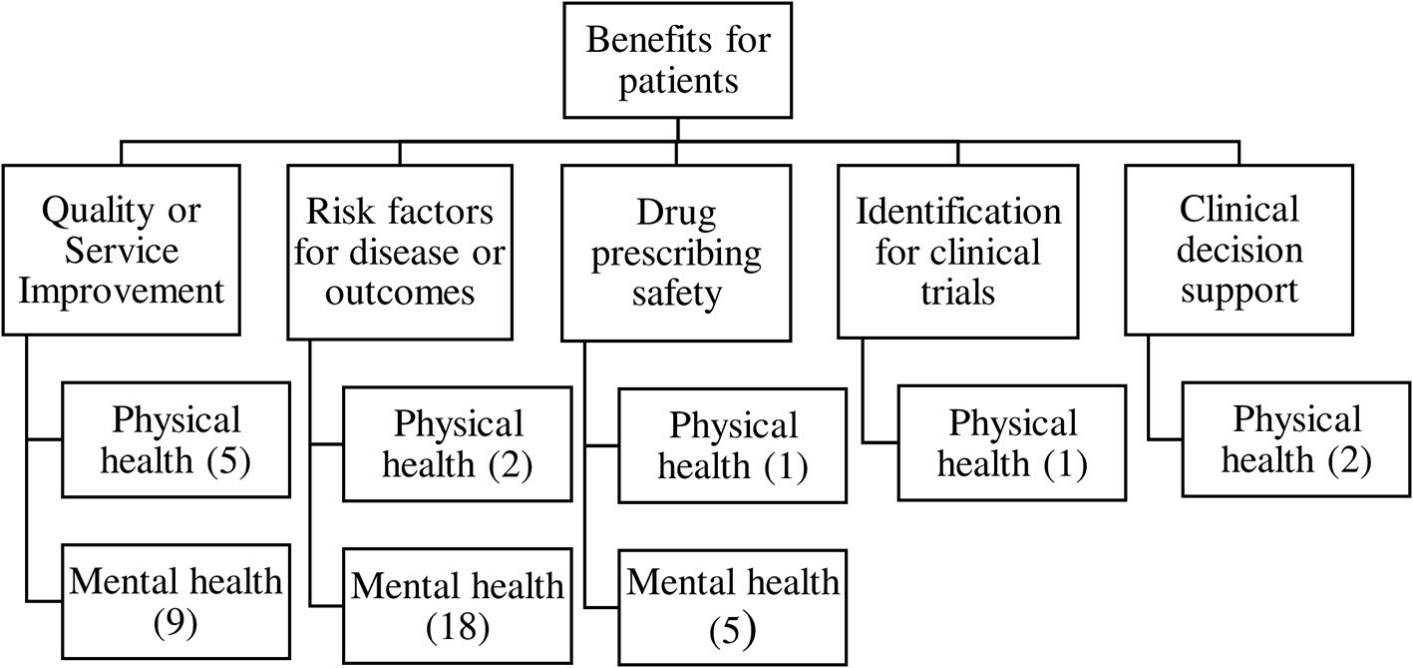
Taxonomy of clinical benefits from analysis of clinical free text data.

Thematic analysis of benefits showed five main types of clinical benefits reported, resulting in the taxonomy shown in Figure 2. The contribution of each study to the themes is shown in Table 1.

**Table 1:** Contribution of each study to themes.

**Theme**	**Health domain**	
	**Physical health**	**Mental health**
Quality or service improvement	Price et al. (46) Ruigómez et al. (82) Tate et al. (48) Woodhead et al. (73) Wu et al. (81)	Bogdanowicz et al. (66) Colling et al. (79) Colling et al. (67) Fusar-Poli et al. (42) Jewell et al. (83) Kadra et al. (69) Leniz et al. (59) Mueller et al. (62) Patel et al. (84)
Risk factors for disease or outcomes	Chang et al. (54) Chang et al. (55)	Das-Munshi et al. (56) Downs et al. (57) Knapp et al. (58) Mansour et al. (60) Mueller et al. (61) Mueller et al. (63) Mukadam et al. (77) Patel et al. (74) Patel et al. (75) Patel et al. (80) Ramu et al. (72) Roberts et al. (65) Sharma et al. (78) Soysal et al. (85) Taylor et al. (43) Taylor et al. (44) Tulloch et al. (45) Tulloch et al. (49)
Drug prescribing safety	Cea-Soriano et al. (41)	Fernandes et al. (6) Kadra et al. (70) Kadra et al. (71) Legge et al. (76) Mueller et al. (64)
Identification for clinical trials	Shah et al. (50)	–
Clinical decision support	Anwar et al. (40) Maguire et al. (47)	–

### Quality or Safety Improvements in Healthcare

Fourteen papers reported healthcare or service quality or safety improvements that were enabled or augmented with the addition of free text information to coded data in health records, five studies focused on physical health (46, 48, 73, 81, 82) mainly drawn from general practice records; and nine on mental health (42, 59, 62, 66, 67, 69, 79, 83, 84). Authors suggested that the increase in information accuracy extracted from health records would result in service improvements such as better service planning due to more accurate prevalence or risk factor estimates, as well as better understanding of symptoms of diseases pre-diagnosis, which might speed up recognition of conditions in primary care. One example is Fusar-Poli et al. (42) who used free text data about people with first episode psychosis to show that they have a better outcome if first seen in services for people at high risk of psychosis before diagnosis, which authors argue may help health trusts to plan and prioritize services. A second example is Woodhead et al. (73) who examined whether breast and cervical cancer screening uptake is lower in women with serious mental illness. Authors stated that understanding this health disparity would help development of policies which encourage “*greater screening uptake among women with other markers of severity or risk, beyond SMI status alone”* (73). A final example is Patel et al. (84) who showed that individuals with bipolar disorder experienced delay in diagnosis and treatment if they had prior diagnoses of alcohol and substance misuse disorders. The authors suggested that these findings would enable “*strategies to better identify underlying symptoms and offer appropriate treatment sooner in order to facilitate improved clinical outcomes”* (84).

### Associations Between Risk Factors and Disease or Disease Outcome

Closely related to the service improvement theme, 20 papers reported associations between exposures or risk factors and risk of disease or clinical outcomes. Two papers reported on physical health outcomes, these assessed the impact of serious mental illness on cancer diagnosis and survival (55), and the impact of intellectual disability on hospital admission and treatment outcomes for severe respiratory diseases (54). A further 18 papers reported on risk factors for mental health disorders and their outcomes (43–45, 49, 56–58, 60, 61, 63, 65, 72, 74, 75, 77, 78, 80, 85). These papers often described risk factors for disease which could enable services to be planned more appropriately to make early diagnosis or meet patients' needs. One example is Taylor et al.'s study of pregnant women who also had severe mental illness (SMI) (43) describing their socio-demographic characteristics and prescribing history, and concluding:

“*A significant proportion of women, particularly those with non-affective psychoses, have modifiable risk factors requiring tailored care to optimize pregnancy outcomes*.” (43)

Authors argue these findings would enable better planning of maternity services to meet the needs of pregnant women with SMI. Several studies looked at risk factors for outcomes of patients with dementia. Knapp et al. identified a range of risk factors for adverse outcomes such as admission to care homes for patients with Alzheimer's disease (58), and Sharma et al. (78) examined predictors of falls and fractures in people with dementia. They concluded:

“*Clinicians should consider that besides established demographic and physical health related factors, the risk of hospitalization due to a fall or fractures in dementia is largely determined by environmental and socioeconomic factors.”*(78)

Soysal et al., used free text data to understand polypharmacy in dementia patients and examined its impact on cognitive decline, finding that:

“*Polypharmacy defined by the number of drugs does not appear to predict cognitive decline in a naturalistic cohort of patients with dementia*” (85).

Taken together these studies give a range of insights of risk factors for developing disease, and for positive and adverse outcomes of diseases which could inform improvements and reduce inequity in healthcare planning and services.

### Drug Prescribing Safety

Six papers reported on associations between prescriptions of drugs and their outcomes, either in terms of adverse drug reactions (ADRs), or in terms of clinical outcomes. One focused on physical health (41), examining the safety of non-insulin drugs for diabetes used during pregnancy. A further five focused on mental health (64, 68, 70, 71, 76). For example, Legge et al. (76) examined reasons for discontinuation of clozapine in patients with schizophrenia. Clozapine is effective but often the last drug to be tried in treatment-resistant schizophrenia and therefore failure of this drug is associated with adverse outcomes for the patient. The study identified that ADRs are an important cause of discontinuation of clozapine and authors suggest these should be treated more aggressively in the treatment onset phase to reduce discontinuation. Other studies examined antipsychotic poly-pharmacy (APP), and risk of readmission to hospital (71) as well as long term outcomes of APP such as mortality risk (70), including several different adjustments for confounding:

“*Our results suggest that patients discharged on APP are more likely to be readmitted into hospital within 6 months in comparison to those discharged on monotherapy. This needs to be considered in treatment decisions*.” (71)

These studies have the potential to change prescribing practice in the clinic, leading to safer or more effective management of patients' conditions.

### Methods for Identifying Patients for Clinical Trials

This theme was sparsely populated by included papers. One paper focused on GP patient records, and identified that inclusion of free text increased the recorded proportion of patients with chest pain in the week prior to MI compared to structured data, and enabled differentiation between MI subtypes (50).

“*Free text contained a large number of records of suspected conditions, for which the clinical system does not provide a facility for structured recording*.” (50)

It is therefore possible to extrapolate from this study that when searching records for potential patients for clinical trials, more patients may be identifiable in the early stages of illness when algorithms have access to the free text in the records, in combination with structured data.

### Development of Clinical Decision Support

Two papers reported on using free text data from patient records to contribute to clinical decision support systems, both papers focused on physical conditions (40, 47). Maguire et al. (47) suggested that the addition of free text to coded data in GP patient records could improve the sensitivity of clinical support systems to help clinicians identify rare diseases, in this case allergic bronchopulmonary aspergillosis (ABPA). The second study, by Anwar et al., used all the data in a patient's record in a hospital audiology department, including free text, to predict what type of hearing aid should be prescribed to the patient (40). Wide adoption of accurate clinical support systems may improve consistency or quality of care for patients in some healthcare clinics.

### Does Inclusion of Free Text Enable Better Quality Research Than Using Structured Data Alone?

While the majority of papers used a combination of analyzing both Read or ICD codes, structured demographic data, and free text information from their respective databases, few reported on the change in quality or accuracy of case identification or data extraction by the addition of free text data.

Most commonly this was reported in papers using GP patient records, where much clinical information is recorded in codes. Five studies using GP patient records reported improved accuracy in detecting symptoms or cases, or that greater clinical information was available for the research study, with the addition of free text (46–48, 50, 82). A further three papers using mental health data reported that more data on variables of interest were available in the free text as compared to structured data, and used free text mentions of diagnoses to augment case finding, but did not quantify the additional patient numbers found by this method (67, 79, 81).

## Discussion

We identified 43 UK studies which reported results of research using clinical free text which would result in direct or potential benefits to patients in terms of informing or improving the quality of their healthcare. The majority of papers used data drawn from patient records in a secondary care mental health trust in South London. This skewed our results to focus in the majority on benefits to patients in a mental health setting in terms of health condition. However, we have no reason to assume this skewed the results in terms of themes identified. We were able to classify the benefits reported in these papers into five clear themes which may aid public audiences in the UK in understanding why researchers want to access free text data from UK-based EHRs.

Our first three themes were focused on issues pertaining to patient care in the clinic and were well-supported with a range of studies; these themes were healthcare quality and service improvements; understanding risk factors for disease and disease outcomes; and improving the safety of drug prescribing. These three themes show the potential for clinical free text to contribute to research which changes clinicians' practice and contributes to the evidence-base for setting up equitable services appropriate to patients' needs, understanding who is at risk of adverse outcomes, and how drugs can be used to best treat symptoms and prevent adverse outcomes.

Two more themes related to the potential development of technology for use in the clinic, one showing case identification methods which illustrated the potential use of free text to contribute to earlier or wider recruitment of patients for clinical trials. This kind of technology may enable more patients to benefit from trial participation, as well as future patients benefiting from higher quality and faster trials, meaning new treatments are available sooner. The other theme showcased examples where free text data could be used to develop automated clinical decision support, helping clinicians make decisions on patients' diagnosis or treatment on the basis of a range of information in their records. These two technology themes were less well-populated than the more clinically focused themes, suggesting the production of well-implemented and integrated healthcare technology which draws on clinical free text may be some way off. However, if we look at wider literature on this topic, including papers which report a purely methodological focus (86, 87), or discuss more widely the future applications of analyzing data from mental health records (88), we can discover more examples of these types of technologies under development.

These themes of clinical benefit are largely in line with other reviews on similar topics. For example, Velupillai et al. (10) also identified mental health as a clinical domain which would richly benefit from the application of NLP to clinical records, and determined that prescribing outcomes, risk factors and prognoses were all features which could be extracted from clinical text and examined in research. Of interest, these authors suggested that the self-reported patient experience could also be examined using clinical free text, but we did not find this theme in our analysis of published papers.

The majority of our papers drew on one UK-based data source, curated by a secondary care trust in south London (SLaM), and focusing on mental health. This reflects that when a safe and secure research infrastructure is developed, allowing NLP researchers to work closely together with clinicians, a very productive and profitable research pipeline can be formed. Of note, SLaM uses a participatory governance model with a service user and carer advisory group which advises researchers on the development of their studies and on requesting linkages (89). This format ensures that research conducted using mental health datasets is directed by priorities identified by service users, that there is a route by which service users can find out about and become involved in research. This reduces the separation between service users and researchers, helping to increase trust. This model of transparency in the use of patient data was favored by members of the Brighton citizens' jury who were asked about their views on sharing their medical free text for research (31).

### Strengths and Limitations

Our review is strengthened by our “cast the net wide” approach to finding papers, by our team approach to sifting, screening and quality-appraising the papers, and by adhering to reporting guidelines. However, we note a few limitations. Our search strategy did not pick up all eligible papers and several were found only after examination of a dataset's published bibliography. This suggests our keywords were not perfectly aligned to the papers we sought, or that papers using free text are not well-labeled as such in their title or abstract. This is something that could be addressed within the research community. Due to our aim being to inform UK-wide discussion, public engagement and policy, we limited our review to UK data only. Therefore, the themes of benefit identified indicate only the historical work that has been possible to conduct in the UK to date. An international search, encompassing differing research groups, health care structures, and data access regulations and policies would likely have yielded additional themes showing types of patient benefit. However, this review still provides a case study of international interest, and which may be used as a resource for policy discussions internationally. Due to the differing rates of progress in accessing free text data between research groups and healthcare settings in the UK, and due to the types of data recording in different part of the health system, the majority of papers so far published in the UK are on mental health. This reflects the UK's early adoption of EHRs in mental health compared to other specialist or hospital care, the national reticence around free text access for primary care EHRs and the current lack of workable governance solutions to free text access outside of mental health. These are influences of national policy with demonstrable impact on the field as reviewed. This will have introduced bias into our understanding of what possible benefits can be accrued from free text data. This bias will not be quantifiable until further work can be done to extend understanding across international research, and until access to free text data is improved in the UK for researchers aiming to study physical health conditions.

This heterogeneous group of studies was also difficult to appraise for quality and for risk of bias across studies. As we were not searching for effect sizes relating to interventions, it was not clear how to evaluate publication bias or selective reporting in our identified body of research.

The derivation of themes for our taxonomy was subjective and certain papers were felt to fit into more than one theme. The two themes of clinical decision support and recruitment to clinical trials were poorly supported by included papers, although they are the focus of methodological work known to the authors. The included papers using GP patient records data were less directly focused on clinical benefits to patients, often reporting data quality improvements as the main outcome of examining free text, although they did suggest potential clinical benefits when discussing their results. Their inclusion was debated among the authors. We aimed to be inclusive so that the maximum number of exemplar studies which described potential benefits could be showcased. Future work should revisit the taxonomy derived here, which may not have reached saturation, and which may be modified by the emergence of future work in this fast developing field.

The majority of studies identified did not present any evaluation of the impact that free text had on the study, as they primarily focused on the clinical benefits. We were unable to categorize the impact that the addition that free text had on improving study sample size or quality or influencing results. Future studies should consider clearly reporting the extent to which free text contributed in a quantifiable way so that this can be evaluated, and studies which provide a standalone assessment of the value of text data for a particular problem should be published alongside.

Finally, while we were able to synthesize potential benefits here, we did not find any reports in the studies on potential harms or costs associated with using free text data. We assume this is because we explicitly excluded papers which evaluated methodology as a primary focus of the study. Our aim is to contribute to discourse on safe and acceptable access to free text clinical data for researchers, and the hope is that when governance structures are developed and implemented, costs and harms of accessing free text data will be minimized for all stakeholders.

### Future Research Directions and Conclusions

We believe these findings will be a useful resource for the public discourse on negotiating access to patient free text data in the UK and provide a methodological template for similar discussions in other international settings. Members of the research community, data-sharing regulators (32) and the public (31) have all expressed a desire to understand more about the potential benefits to health of sharing free text data from clinical records, and this review offers the first resource where a body of relevant evidence has been pulled together. As a next step, we intend to set up an online repository through an openly available website (such as www.healtex.org), which will list the papers identified in this review and give a short summary of expected patient benefit from the research for each one. This resource could be “live” and continuously updated. Open access approaches to dissemination such as project websites can be excellent ways of communicating with a range of stakeholders. We hope these results will contribute to widespread understanding of the value of using clinical free text in research to improve the health and healthcare of the UK population, and to progressive data governance regulation which works for the good of the whole population.

## Data Availability Statement

The original contributions presented in the study are included in the article/Supplementary Materials, further inquiries can be directed to the corresponding author.

## Author Contributions

EF conceived the project, screened articles, analyzed data, and drafted the manuscript. KC conducted searches, screened articles, extracted data, analyzed data, and helped to draft the manuscript. KJ, ES, and LG screened articles, extracted data, and commented on drafts of the manuscript. RS commented on drafts of the manuscript. All authors read and approved the final manuscript.

## Conflict of Interest

The authors declare that the research was conducted in the absence of any commercial or financial relationships that could be construed as a potential conflict of interest.

## Supplementary Material

Appendix 1 Joanna Briggs Institute critical appraisal checklist screening results.

Appendix 2 PRISMA checklist.

## Appendix

**Table A1:** Overview of included studies.

**References**	**Clinical research question(s)**	**Data source and type**	**Number of patients and/or documents included in analysis**	**Free text information extraction method and purpose**	**Summary of clinical findings**	**Statement of potential clinical impact**	**Theme of benefit**
Anwar et al. (40)	To investigate if a decision support system be produced using EHRs to predict type of hearing aid prescription.	Hospital patient records; including audiograms categorical data and free text notes	Over 23,000 patients Over 180,000 documents.	Manual extraction of notes made by hearing aid technicians.	The decision support system was able to replicate the decisions of audiologists whether to fit an in the ear or a behind the ear hearing aid with high precision.	A decision support system was produced to predict prescription of different hearing aids alongside an explanation demonstrating how that decision was arrived at. Authors conclude this will provide a useful “second opinion” for audiologists.	Clinical decision support
Bogdanowicz et al. (66)	To investigate clustering of all-cause and overdose deaths after a transfer of patients to alternative treatment provider and after the end of opioid substitution therapy (OST) in opioid-dependent individuals in specialist addiction treatment.	South London and Maudsley (SLaM) Biomedical Research Center (BRC) Case Register using Clinical Records Interactive Search (CRIS)	5,335 patients and 216 documents.	NLP algorithms to extract diagnoses, start and end dates of treatment episodes, medication and reasons for treatment cessation from text.	Opioid-dependent people who are transferred to an alternative treatment provider for continuation of their opioid substitution therapy experience high overdose mortality rates, with substantially higher rates during the first month.	Any transfer of patients whether due to escalation of treatment (e.g., to an in-patient unit) or as part of successful recovery (e.g., from an in-patient unit to rehabilitation care) needs to be undertaken with caution.	Service improvement
Cea-Soriano et al. (41)	To investigate if there is an association between the use of non-insulin antidiabetics in early pregnancy and the risk of miscarriages, stillbirths and major structural malformations.	The Health Improvement Network (THIN)	1,511 pregnancies in women with pre-gestational diabetes linked to livebirths; one full record per patient.	Manual extraction of free text to identify infant malformations	In pregnant woman with diabetes, use of non-insulin antidiabetic agents in early pregnancy had no link with greater risks of fetal losses or major malformations than insulin.	Adverse outcomes to infants born to women with type 1 diabetes can be predicted and prevented by the analysis of EHRs including free text.	Drug prescribing safety
Chang et al. (54)	To assess how intellectual disability (ID) affects the risks of severe respiratory diseases leading to hospital admission and how much worse their treatment outcomes are by comparing critical indicators for service usage	SLaM BRC CRIS Case Register linked to Hospital Episodes Statistics (HES)	3,138 patients; one full record per patient.	Generalized Architecture for Text Engineering (GATE) for NLP to supplement diagnostic codes for case identification.	Respiratory system disease admissions in adults with ID are more frequent, of longer duration and have a higher likelihood of recurring.	More intervention studies are needed to generate prevention strategies specifically for respiratory system diseases in primary and secondary care for adults with ID.	Risk factors for disease & outcomes
Chang et al. (55)	To assess the stage at cancer diagnosis and survival after cancer diagnosis among people served by secondary mental health services, compared with other local people.	SLaM BRC CRIS Case Register linked to Thames Cancer Registry	28,477 patients; one full record per patient	GATE for NLP to supplement diagnostic codes for case identification.	No link was found between mental disorder diagnoses and spread of cancer at presentation. However, people with severe mental disorders, depression, dementia and substance use disorders had significantly worse survival after cancer diagnosis, independent of cancer.	EHRs can be used in observational research to identify outcomes of individuals with mental disorders and other co-morbidities, such as cancer.	Risk factors for disease & outcomes
Colling et al. (79)	To investigate what proportion of patients with psychosis received CBT as compared to published audits and explore whether demographics predicted receiving CBT.	SLaM BRC CRIS case register	2,579 and 2,308 service users from two published audits, one full record per patient.	NLP techniques to identify episodes of CBT for psychosis.	Younger, white patients with a diagnosis of other schizophrenia spectrum and schizoaffective disorder when compared with schizophrenia were significantly more likely to have received CBT. Free text analysis improved accuracy of identifying patients.	The method provides a useful way for evaluating delivery of CBT in persons with psychosis on a large scale. It provides quality and safety service improvement by providing more scope for routine monitoring, and the promotion of equitable access between different demographic groups.	Service improvement
Colling et al. (67)	To investigate whether EHRs predict priority service outcomes in a mental health setting and whether NLP enhance these predictions, through the evaluation of three outcomes: (1) inpatient duration (2) readmission following inpatient care (3) high-intensity service use following first referral	SLaM BRC CRIS case register	(1) 808 patients (2)1,650 patients (3) 4,494 patients, one full record per patient.	NLP using TextHunter to develop algorithms on GATE for symptoms and medications	For extended duration of hospital admission and readmission following hospital admission (outcomes 1 and 2), predictive models did not achieve adequate levels of performance. Predicting high-intensity service use following a first referral showed promisingly sustained performance from development to evaluation data. NLP improved the predictions	EHRs can lead to quality service improvement by improving routine clinical predictions through utilizing previously inaccessible data.	Service improvement
Das-Munshi et al. (56)	To estimate excess mortality for people with severe mental illness across different ethnic groups, and to assess the association of ethnicity with mortality risk.	SLaM BRC CRIS case register with linked mortality data from Office of National Statistics (ONS)	18,201 patients; one full record per patient.	NLP techniques using GATE to supplement diagnosis codes for case identification.	People with severe mental illness have a higher excess mortality relative to the general population regardless of ethnicity. Among those with severe mental illness, some ethnic minorities have lower mortality than the white British group, for which the reasons deserve further investigation.	EHRs can be used in observational research to investigate exposure-outcome association in large mental healthcare setting.	Risk factors for disease & outcomes
Downs et al. (57)	To investigate the prevalence of negative symptoms (NS) at first episode of early-onset psychosis (EOP), and their effect on psychosis prognosis.	SLaM BRC CRIS case register	1,033 patients, 618 records	NLP techniques using GATE to supplement diagnostic codes for case identification, identify antipsychotic prescriptions, identification of multiple treatment failure.	Findings support the hypothesis that presence of these symptoms around the first stages of the illness identify a subset of children and adolescents who may be at higher risk of responding poorly to antipsychotics, both through refractory symptoms and high sensitivity to side-effects	Optimization of current pharmacological and non-pharmacological strategies for these patients, and further research involving agents that better target NS are warranted.	Risk factors for disease & outcomes
Fernandes et al. (68)	To investigate the demographic and clinical factors associated with antidepressant use for depressive disorder in a psychiatric healthcare setting	SLaM BRC CRIS case register	1,561 patients; one full record per patient.	NLP techniques using GATE to identify medications	Age, past medication and/or psychotherapy receipt use and symptom profiles in the past 12 months were associated with antidepressant receipt in secondary mental healthcare.	Continual monitoring of treatment choices in this cohort may contribute to providing optimal care for secondary care patients	Drug prescribing safety
Fusar-Poli et al. (42)	To compare the clinical outcomes in first episode of psychosis (FEP) patients who presented to either high risk or conventional mental health services.	SLaM BRC CRIS case register	2,943 patients; one full record per patient.	Manual review of free text for case identification.	FEP patients who had presented to a high-risk service spent fewer days in hospital, had a shorter referral to-diagnosis time, a lower frequency of admission and a lower likelihood of compulsory admission in the 24 months following referral, as compared to FEP patients who were first diagnosed at conventional services.	This study provides the first evidence that services designed for people at high risk of psychosis may be associated with better outcomes in patients who are already psychotic, but were referred because they were thought to be at high risk.	Service improvement
Jewell et al. (83)	To determine whether dynamic factors related to behavior, cooperation with treatment, and activities on the ward are associated with outcome at Mental Health Review Tribunals (MHRTs) in forensic psychiatric patients.	SLaM BRC CRIS case register	79 patients; one full record per patient.	NLP techniques using GATE to identify violence risk assessment score.	Unescorted community leave, responsible clinician's recommendation of discharge, and restricted Mental Health Act section were associated were greater likelihood of discharge at tribunal. Inpatient aggression, recent episode of acute illness, higher scores on violence risk assessment and agitated behavior were all negatively associated with discharge at tribunal. Historical Clinical Risk scores and attempted or actual physical violence uniquely predicted outcome after controlling for other dynamic variables.	By identifying dynamic factors in EHRs associated with discharge at tribunal, the results have important implications for forensic psychiatric patients and their clinical teams to improve their chances of discharge at a MHRT	Service improvement
Kadra et al. (69)	This study aimed to investigate socio-demographic, socioeconomic, clinical, and service-use predictors of long-term	SLaM BRC CRIS case register	6,857 patients; one full record per patient.	NLP techniques using GATE to identify medication prescribing and supplement diagnosis codes.	Findings highlight that certain patient groups are at an increased risk for long-term APP initiation.	Identifying the patients who are at an increased risk for long-term APP treatment earlier in their treatment could encourage clinicians to employ a broader range of interventions in addition to pharmacotherapy to reduce the risk of APP prescribing.	Service improvement
		APP initiation in serious mental illness (SMI).					
Kadra et al. (70)	The aim of this study was to determine if there was an association between being discharged on antipsychotic polypharmacy (APP) and risk of readmission into secondary mental health care.	SLaM BRC CRIS case register	5,523 patients; one full record per patient.	NLP techniques using GATE to supplement antipsychotic medications and diagnostic data and to identify medication non adherence.	Being discharged on APP was associated with a significantly increased risk of readmission, in comparison to patients discharged on monotherapy. Patients receiving clozapine antipsychotic polypharmacy (APP) were at a significantly increased risk for readmission in comparison to patients on clozapine monotherapy.	Patients discharged on APP are more likely to be readmitted into hospital within 6 months in comparison to those discharged on monotherapy. This needs to be considered in treatment decisions and the reasons for the association clarified.	Drug prescribing safety
Kadra et al. (71)	To investigate the association between long-term antipsychotic polypharmacy use and mortality; and determine whether this risk varies by cause of death and antipsychotic dose	SLaM BRC CRIS case register linked with ONS mortality data	10,945 patients; one full record per patient.	NLP techniques using GATE to identify medication data including dose, and comorbid diagnoses	Patients on long-term antipsychotic polypharmacy (APP) had a small elevated risk of mortality, which was significant in some but not all models.	The findings suggest that the effect of long-term APP on mortality is not clear-cut, with limited evidence to indicate an association, even after controlling for the effect of dose	Drug prescribing safety
Knapp et al. (58)	To explore the relationships between clinical and other characteristics of people with Alzheimer's disease living in the community and likelihood of care home or hospital admission, and associated costs.	SLaM BRC CRIS case register linked to HES	3,075 patients; one full record per patient.	NLP techniques using GATE to supplement diagnosis codes for case identification, identify MMSE score and care home admission.	The authors were able to predict probability of care home or hospital admission and/or associated costs over 6 months.	EHRs can be used in observational research to predict exposures, such as clinical and demographic characteristics with outcomes, such as destination of care, in a mental healthcare setting.	Risk factors for disease & outcomes
Legge et al. (76)	To investigate the risk factors, reasons and timing of clozapine discontinuation in patients with treatment-resistant schizophrenia (TRS).	SLaM BRC CRIS case register	316 patients; one full record per patient.	NLP techniques using GATE to identify clozapine use and reasons for discontinuation	Adverse drug reactions (ADRs) accounted for over half of clozapine discontinuation. Sedation was the most common ADR cited as a reason for discontinuation and the risk of discontinuation due to ADRs was highest in the first few months of clozapine treatment. High levels of deprivation in the neighborhood where the patient lived were associated with increased risk of clozapine discontinuation.	It is important that clinicians identify and treat ADRs attributed to clozapine, particularly in the first few months after treatment onset, before they lead to discontinuation. Patients who live in an area of high deprivation are at an increased risk of discontinuing clozapine and may need additional support to maintain engagement with treatment.	Drug prescribing safety
Leniz et al. (59)	To investigate determinants of end-of-life hospital transitions, and association with healthcare use, among people with dementia.	SLaM BRC CRIS case register linked to ONS mortality data	8,800 patients; one full record per patient.	NLP techniques using GATE to supplement dementia identification and MMSE score.	Early transitions were associated with more hospital admissions throughout the last year of life compared to those with late and no transitions.	In contrast to late transitions, early transitions are associated with higher healthcare use and characteristics that are predictable, indicating potential for prevention.	Service improvement
Maguire et al. (47)	To evaluate whether primary care EHRs from patients with severe asthma can be used to identify allergic bronchopulmonary aspergillosis (ABPA) cases.	Clinical Practice Research Datalink (CPRD)	21,054 patients; one full record per patient.	Keyword search and manual check of free text to identify ABPA diagnosis, symptoms tests and treatment.	From the observed concurrence of keywords, the authors were able to devise an algorithm to identify cases of ABPA with varying degrees of specificity. Inclusion of free text improved identification of ABPA.	Clinical Decision Support tools can be produced from EHRs to identify ABPA cases in severe asthmatics. This tool could be extrapolated identify other rare conditions and to quantify their potential burden.	Clinical decision support
Mansour et al. (60)	To compare symptoms and types of treatment between ethnic groups in patients with late-life depression.	SLaM BRC CRIS case register	5,546 patients; one full record per patient.	NLP techniques using GATE to supplement diagnosis codes for case identification, identify depressive symptoms, CBT and medications.	Black Africans and Black Caribbeans more frequently presented with psychotic problems and were significantly less likely to have anti-depressant treatment prescribed post-diagnosis compared to White British.	Ethnic minority elders have significantly different presentations and undertake different types of treatment both across groups and relative to their White British counterparts. These differences need to be taken into consideration to optimize pathways into care and to personalize treatment.	Risk factors for disease & outcomes
Mueller et al. (61)	To investigate associations between acetylcholinesterase inhibitors (AChEI) prescription and mortality in patients with Alzheimer's dementia (AD) in a naturalistic setting.	SLaM BRC CRIS case register linked to HES and ONS mortality data	3,199 patients; 2,464 records.	NLP techniques using GATE to supplement diagnosis codes for case identification, medication prescriptions, MMSE score	A strong association between AChEI receipt and lower mortality was observed.	In a large cohort of patients with AD, AChEI prescription was associated with reduced risk of death by more than 20% in adjusted models. This has implications for individual care planning and service development.	Risk factors for disease & outcomes
Mueller et al. (62)	To describe the risk and duration of hospital admissions in patients with dementia with Lewy bodies (DLB), and compare these to those in Alzheimer's disease (AD) and the general population	SLaM BRC CRIS case register linked to HES data	10,159 patients; 970 records.	NLP techniques using GATE to supplement diagnosis codes for case identification.	Patients with DLB are more frequently admitted to general hospitals and utilize inpatient care to a substantially higher degree than patients with AD or the general elderly population.	This data highlights an opportunity to reduce hospital days by identifying DLB earlier and providing more targeted care.	Service Improvement
Mueller et al. (63)	To investigate associations between polypharmacy and emergency department attendance, any and unplanned hospitalization, and mortality in patients with dementia.	SLaM BRC CRIS Case Register linked to HES and ONS mortality data.	4,668 patients; one full record per patient.	NLP techniques using GATE to supplement dementia diagnosis codes for case identification and identify polypharmacy and MMSE score.	Polypharmacy is associated with an increased risk of emergency department attendance, and both any and unplanned hospitalization, as well as mortality. There is also a dose-response relationship between number of medications prescribed and negative outcomes.	Increased risks associated with polypharmacy may have considerable public health and healthcare delivery consequences.	Risk factors for disease & outcomes
Mueller et al. (64)	To investigate the association between use of antidepressants and mortality in people diagnosed Alzheimer's disease (AD).	SLaM BRC CRIS Case Register linked to HES and ONS mortality data.	5,473 patients; one full record per patient.	NLP techniques using GATE to supplement diagnosis codes for case identification, medication and MMSE score	Prescription of an antidepressant, before or after dementia diagnosis, was associated with higher mortality. Risks remained significant in patients without neuropsychiatric symptoms.	Prescription of antidepressants around time of dementia diagnosis may be a risk factor for mortality.	Drug prescribing safety
Mukadam et al. (77)	To investigate interethnic differences in cognitive scores and age at dementia diagnosis.	SLaM BRC CRIS case register	13,789 patients; one full record per patient.	NLP techniques using GATE to supplement MMSE score	In general, ethnic group distributions in referrals did not differ substantially from those expected in the catchments	People from black and Asian groups were younger at dementia diagnosis and had lower MMSE scores than white referrals.	Risk factors for disease & outcomes
Patel et al. (74)	To identify negative symptoms of patients with schizophrenia and assess their relationship with clinical outcomes.	SLaM BRC CRIS case register	7,678 patients; one full record per patient.	NLP techniques using GATE to identify negative symptoms.	Negative symptoms were common and associated with adverse clinical outcomes, consistent with evidence that these symptoms account for much of the disability associated with schizophrenia.	Observational research can be conducted using EHRs in large representative samples of patients, using data recorded during routine clinical practice.	Risk factors for disease & outcomes
Patel et al. (75)	To assess the impact of mood instability on clinical outcomes in patients with a psychotic, affective or personality disorder.	SLaM BRC CRIS case register	27,704 patients; one full record per patient.	NLP techniques using GATE & TextHunter to identify mood instability.	Mood instability occurs in a wide range of mental disorders and is not limited to affective disorders. Mood instability is associated with poor clinical outcomes. Clinicians should screen for mood instability across all common mental health disorders.	Observational research can be conducted in a mental healthcare setting using EHR's to determine the impact of mood instability on patient outcomes.	Risk factors for disease & outcomes
Patel et al. (84)	To investigate factors associated with the delay before diagnosis of bipolar disorder and the onset of treatment in secondary mental healthcare	SLaM BRC CRIS case register	1,364 patients; one full record per patient	NLP techniques using GATE to supplement diagnosis and medication information.	Some individuals experienced a significant delay in diagnosis and treatment of bipolar disorder after initiation of specialist mental healthcare, particularly those who had prior diagnoses of alcohol and substance misuse disorders.	These findings highlight a need for further study on strategies to better identify underlying symptoms and offer appropriate treatment sooner in order to facilitate improved clinical outcomes, such as developing specialist early intervention services to identify and treat people with bipolar disorder.	Service improvement
Patel et al., (80)	To investigate whether cannabis use is associated with increased risk of relapse, and whether antipsychotic treatment failure may mediate this effect in patients with first episode psychosis (FEP).	SLaM BRC CRIS case register	2,026 patients; one full record per patient.	NLP techniques using GATE & TextHunter to identify cannabis use	The use of cannabis in patients with FEP was associated with an increased likelihood of hospital admission and antipsychotic treatment failure did not mediate this.	EHRs can be used to conduct observational research in a mental healthcare capacity, for example to determine that cannabis use (exposure) is associated with various outcomes (relapse risk and antipsychotic treatment failure).	Risk factors for disease & outcomes
Price et al., (46)	To estimate alarm symptoms for cancer recorded in free text.	Clinical Practice Research Datalink (CPRD)	4,915 bladder and 3,635 pancreatic cancer cases; 16,459 control group EHRs; one full record per patient.	Keyword search for symptoms followed by algorithm run in Stata.	Omission of text records from CPRD studies introduces bias that inflates outcome measures for recognized alarm symptoms.	The addition of free text when analyzing EHRs improves accuracy and therefore results in potential improved understanding of symptoms and improvement in recognition of cancer symptoms in general practice.	Service improvement
Ramu et al. (72)	To investigate recorded poor insight in relation to mental health and service use outcomes in a cohort with first-episode psychosis.	SLaM BRC CRIS case register	2,026 patients; one full record per patient.	NLP techniques using GATE & TextHunter to identify poor insight, cannabis use, and antipsychotic prescription.	Recorded poor insight in people with recent onset psychosis predicted subsequent legally enforced hospitalizations and higher number of hospital admissions, number of unique antipsychotics prescribed and days spent hospitalized.	Improving insight might benefit patients' course of illness as well as reduce mental health service use	Risk factors for disease & outcomes
Roberts et al. (65)	To investigate mortality in individuals diagnosed with chronic fatigue syndrome (CFS).	SLaM BRC CRIS case register linked ONS mortality data.	2,147 patients; one full record per patient	NLP techniques using GATE & TextHunter to supplement CFS diagnosis codes for case identification	All-cause and cancer-specific mortality of patients with chronic fatigue syndrome in specialist care is not significantly different to that of the general population, however the risk of suicide is higher	Clinicians need to be aware of the increased risk of completed suicide and to assess suicidality adequately in patients with chronic fatigue syndrome.	Risk factors for disease & outcomes
Ruigómez et al. (82)	To evaluate the validity of recorded diagnoses of ischemic cerebrovascular events requiring hospitalization	The Health Improvement Network (THIN)	4,239 patients; one full record per patient.	Manual extraction of free text to validate codes for cerebrovascular events.	THIN demonstrates a high validity for the study of ischemic cerebrovascular events when reviewing computer records with additional free text comments.	EHRs, especially with the addition of free text comments, are an efficient approach to ascertain the accuracy and validity of computerized data and lead to service improvement.	Service improvement
Shah et al. (50)	To describe the contribution of free text in primary care to the recording of information about myocardial infarction (MI), including subtype, left ventricular function, laboratory results and symptoms; and recording of cause of death.	Clinical Practice Research Datalink (CPRD)	2,000 patients with MI and 1,800 deaths; one full record per patient.	Freetext Matching Algorithm (FMA) NLP software to determine subtype of MI, left ventricular function, angiogram results and symptoms.	Inclusion of free text increased the recorded proportion of patients with chest pain in the week prior to MI from 19 to 27%, and differentiated between MI subtypes in a quarter more patients than structured data alone.	Natural language processing to convert this information into a structured form can enrich primary care data at scale for research, and yield population-based insights into early presentations of disease.	Case finding for clinical trials
Sharma et al. (78)	Investigate predictors of falls and fractures leading to hospitalization in a large cohort of people with dementia.	SLaM BRC CRIS case register linked to HES	8,036 patients; one full record per patient	NLP techniques using GATE to supplement medications and MMSE score	Medications (including psychotropic and antipsychotics), neuropsychiatric symptoms, cognitive (Mini-Mental State Examination scores), or functional problems did not predict hospitalized falls.	Clinicians should consider that besides established demographic and physical health related factors, the risk of hospitalization due to a fall or fractures in dementia is largely determined by environmental and socioeconomic factors.	Risk factors for disease & outcomes
Soysal et al. (85)	To test associations between polypharmacy and both short-term (6 months) and long-term (3 years) cognitive trajectories in patients with incident dementia.	SLaM BRC CRIS case register	12,148 patients; one full record per patient.	NLP techniques using GATE to identify dementia date of diagnosis and number of medications.	No significant differences to the control group were found in relation to polypharmacy or excessive polypharmacy, neither in the initial cognitive improvement nor long-term decline.	Polypharmacy defined by the number of drugs does not appear to predict cognitive decline in a naturalistic cohort of patients with dementia.	Risk factors for disease & outcomes
Tate et al. (48)	To investigate how much information on ovarian cancer diagnosis is ‘hidden' in the free text and the time lag between a diagnosis being described in the text or in a hospital letter and the patient being given a Read code for that diagnosis.	Clinical Practice Research Datalink (CPRD)	344 patients; one full record per patient.	Manual extraction of information on date of diagnosis	Free text contains a significant amount of extra information. Free text analysis showed that GPs do not always code a definite diagnosis on the date that it is confirmed. For diseases which rely on hospital consultants for diagnosis, free text (particularly letters) is invaluable for accurate dating of diagnosis and referrals.	EHRs using free text can be used to improve the amount of information available to clinicians, leading to improved quality of care.	Service improvement
Taylor et al. (43)	To investigate the socio-demographic and clinical characteristics of an epidemiologically representative cohort of pregnant women with affective and non-affective severe mental illness.	SLaM BRC CRIS case register linked with HES	456 patients; one full record per patient.	NLP techniques using GATE to supplement diagnosis codes for case identification, number of days in acute mental health care. Manual searches for expected delivery dates, number of other children, partner status, smoking, alcohol, drug use, maternal history of abuse, deliberate self-harm.	A significant proportion of women, particularly those with non-affective psychoses, have modifiable risk factors requiring tailored care to optimize pregnancy outcomes. Mental health professionals need to be mindful of the possibility of pregnancy in women of childbearing age and prescribe and address modifiable risk factors accordingly.	EHRs can be used for observational research by predicting risk factors (exposures) for pregnancy outcomes (outcome).	Risk factors for disease & outcomes
Taylor et al. (44)	To investigate risk factors for postpartum relapse, particularly the potential prophylactic effects of medication in women with non-affective or affective psychoses.	SLaM BRC CRIS case register linked to HES	452 patients; one full record per patient.	NLP techniques using GATE to supplement diagnosis codes for case identification, and medication exposure. Manual searches for expected delivery dates, number of other children, partner status, smoking, maternal history of abuse, family history of psychosis, smoking, drug and alcohol use.	There was no evidence of a prophylactic effect of medication in women with non-affective or affective psychoses	Recent relapse increases the risk of relapse in the postpartum period so women with severe illnesses with a recent history of relapse should be warned pre-conception about the high risk of relapse.	Risk factors for disease & outcomes
Tulloch et al. (45)	To investigate the associations of homelessness and residential mobility with length of stay after acute psychiatric admission	SLaM BRC CRIS case register	4,885 patients; one full record per patient.	Housing data supplemented by manual review of free text for homelessness status.	Homelessness and, especially, residential mobility account for a significant part of variation in length of stay despite affecting a minority of psychiatric inpatients; for these people, the effect on length of stay is marked.	Residential mobility and, to a lesser extent, homelessness appeared to have very significant effects on length of stay which should inform policy change. The effects are clearly detectable within the whole sample, but apply especially to the minority of individuals who are directly affected.	Risk factors for disease & outcomes
Tulloch et al. (49)	To investigate the clinical and demographic associations of khat use in a sample of Somali users of mental health service users in South London.	SLaM BRC CRIS case register	240 patients; one full record per patient	Keyword search plus manual coding of Somali origin, Khat, cannabis and alcohol use, and diagnoses.	Khat use was very strongly associated with a primary diagnosis of schizophrenia, psychosis or drug and alcohol disorder (compared to stress-related disorders and other non-psychotic disorders), male gender, harmful or dependent use of alcohol, and detention under the Mental Health Act.	Documentation of khat use should be improved, especially for patients of Somali, Yemeni and other relevant ethnicity.	Risk factors for disease & outcomes
Woodhead et al. (73)	To examine if breast and cancer screening uptake is lower among women with serious mental illness (SMI) and to identify variation in screening uptake by illness/treatment factors, and primary care consultation frequency.	SLaM BRC CRIS case register linked to Lambeth DataNet (LDN);	Patients with breast and cervical screening receipt among linked eligible SMI patients (*n* = 625 and *n* = 1,393), to those without SMI known only to primary care (*n* = 106,554 and *n* =25,385); one full record per patient	NLP techniques using GATE to identify antipsychotic use, and risk and severity variables.	Women with SMI are less likely to receive breast and cervical cancer screening than comparable women without SMI.	To tackle health disparities linked to SMI, efforts at increasing screening uptake are key and should be targeted at women with other markers of illness severity or risk, beyond SMI status alone.	Service improvement
Wu et al. (81)	To investigate the prevalence and correlates of EHR-derived current smoking in people with severe mental illness.	SLaM BRC CRIS case register	1,555 patients; one full record per patient.	NLP techniques using GATE to identify smoking status	Proportions of patients with recorded smoking status increased from 11.6 to 64.0% when using CRIS data. After adjustment, younger age (below 65 years), male sex, and non-cohabiting status were associated with current smoking status.	A natural language processing application substantially improved routine EHR data on smoking status above structured fields alone and could thus be helpful in improving monitoring of this lifestyle behavior.	Service improvement
